# Socio-economic predictors of public understanding of the COVID-19 pandemic

**DOI:** 10.1016/j.heliyon.2021.e07255

**Published:** 2021-06-09

**Authors:** Md Rifat Hossain, Salit Chakma, Farah Tasnim, Zuairia Zahra

**Affiliations:** Department of Disaster and Human Security Management, Bangladesh University of Professionals, Bangladesh

**Keywords:** COVID-19, Awareness, Socio-economic factors, Behavioral pattern, Mental condition, Ordered logit model

## Abstract

The COVID-19 (Coronavirus 2019) pandemic has proven to be the biggest global shock since World War II. That war resulted in 5.5 million deaths. The number of COVID-19-infected persons exceeded 13 million in the first 6 months of the pandemic and many more asymptomatic cases are undocumented. The global economy has been affected severely. The tension, the fear, the drastic measures to try to control the spread of the disease disrupted everyone's life from child to senior. The condition is worse in the global south, such as in Bangladesh, where the average population density is 7.5 times higher than that of China, where COVID-19 began and spread uncontrollably at the end of 2019. Lockdowns and social distancing were tried to stop the transmission of the disease but were often not observed faithfully or were less effective than thought to be. People need to trade and interact to earn money to survive but these activities could endanger others' lives if they do not maintain safety measures. Individual awareness is not only curtailing the spread of COVID-19 but also saves others' lives. This cross-sectional study used Ordinal and Binary logit models to predict the level of awareness through potential regressors of the citizen toward COVID-19 in Bangladesh. Findings of the study are that the level of awareness is dependent on the level of trauma; also, that household income is a statistically-significant predictor of awareness. Behavioral activities such as use of masks, outdoor activities, and stockpiling tendencies are found to be statistically significant predictors of awareness as well.

## 1 Introduction

The COVID-19 virus is an unfamiliar and advanced strain of coronavirus (CoV), identified by Chinese scientists on January 09, 2020. By April 2020, this coronavirus had given rise to a global pandemic (Dryhurst et al., 2020).

Coronavirus induces illnesses ranging from common cold to acute respiratory diseases similar to previous epidemics, such as, Middle East Respiratory Syndrome (MERS-CoV) and Severe Acute Respiratory Syndrome (SARS-CoV) (Shaw et al. 2020). A strand of this virus was previously recorded in humans for the first time in 2003 in China (Alam et al., 2020). The COVID-19 coronavirus is the third new pathogen to appear in the last two decades (Al-Hazmi 2016). Coronavirus is transmitted from person to person through small droplets produced while coughing, sneezing, talking, and even by touching contaminated surfaces. Although the contamination rate is very high, the mortality rate is only 2.3% much less than that of Ebola or SARS (Qazi et al., 2020; Zhong et al., 2020).

Travel and movement rapidly spread the virus over 206 countries, with 900,306 confirmed cases and 45,693 deaths globally by April 02, 2020 (Paul et al. 2020). China locked down from January 13, 2020 to try to stop the spread of the disease. On the same day, WHO reported a case in Thailand, making it the first country to have a corona-positive patient after China. By January 17, 2020, the U.S.A., Nepal, France, Australia, Malaysia, Taiwan, Singapore, and Vietnam had confirmed their first cases of COVID-19 (Lai et al., 2020). Italy was the first European country to impose a quarantine to attempt to control the influx of COVID-19. The south Asian countries (India, Pakistan, Sri Lanka, Nepal, Bhutan and Bangladesh) faced extreme difficulties in trying to make the illiterate masses follow social distancing and quarantine due to poverty and density of populations (Djalante et al. 2020; Paul et al. 2020).

Many countries initiated preventive measures, including work from home, lockdown, hygiene campaigns, and social distancing to limit transmission (Anwar et al. 2020). Gradually, international travel was immobilized, and economic activities came to a standstill as a result of lockdowns in country after country. When the World Health Organization (WHO) declared COVID-19 a pandemic on March 11 (Hua and Shaw 2020; Lai et al., 2020), it triggered several unprecedented changes in socio-economic behavior. The uncertainty related to public safety and misleading information about COVID-19 had an adverse impact on individuals’ mental health, resulting in depression and psychological trauma (Mamun and Griffiths 2020; Nicola et al., 2020; Roy et al., 2020). Negative feelings and behaviors increased together, including domestic abuse and substance addiction. Li et al. (2020) explained that the roots of these adverse feelings emanate from the perceived need for “self-protection”.

### 1.1 First detection, gradual transmission, and impact in Bangladesh

Overcrowding, inadequate health care facilities, limited resources, and poverty have facilitated rapid community transmission of COVID-19 since its first detection in Bangladesh (Shammi et al., 2020). On March 8, 3 Bangladeshi were found COVID-19 positive (Alam et al., 2020). Bangladesh's Government soon set up temporary quarantine at Haji Camp (in Hazrat Shahjalal International Airport) for oversees returnees (Islam and Siddika 2020). Although the Government encouraged expatriates to do home quarantine, some of the returnees were found roaming around, ignoring the Government guidelines (Anwar et al. 2020). They were given fines ranging from $35.34 to $589.08 (‘First coronavirus cases confirmed’ 2020). Bangladesh's Government locked the country down from late March, arguing it would gradually stop the outbreak (‘Fighting Coronavirus: Govt Shuts Down Offices from Mar 26’ 2020). Meanwhile, insufficient health facilities put a strain on the psychology of the people (Banik et al., 2020). Despite a massive public awareness campaign, a mass prayer assembly of approximately 25,000 people were held on March 18, flouting local authorities' prohibitions (Anwar et al. 2020). Many convened with a faith that this prayer would safeguard them from the virus. A month later, on April 18, approximately 100,000 people gathered for funeral of a renowned religious leader, defying the orders of law enforcement (Shammi et al., 2020). Many claimed the unofficial death toll to be higher than the reported figure, as many people did not receive treatment and were never counted or hide their illness to avoid social stigma (Dubey et al., 2020). Thus, we can see that the lack of awareness among citizens is the primary factor that facilitated the outbreak in the country.

**Figure 1:**
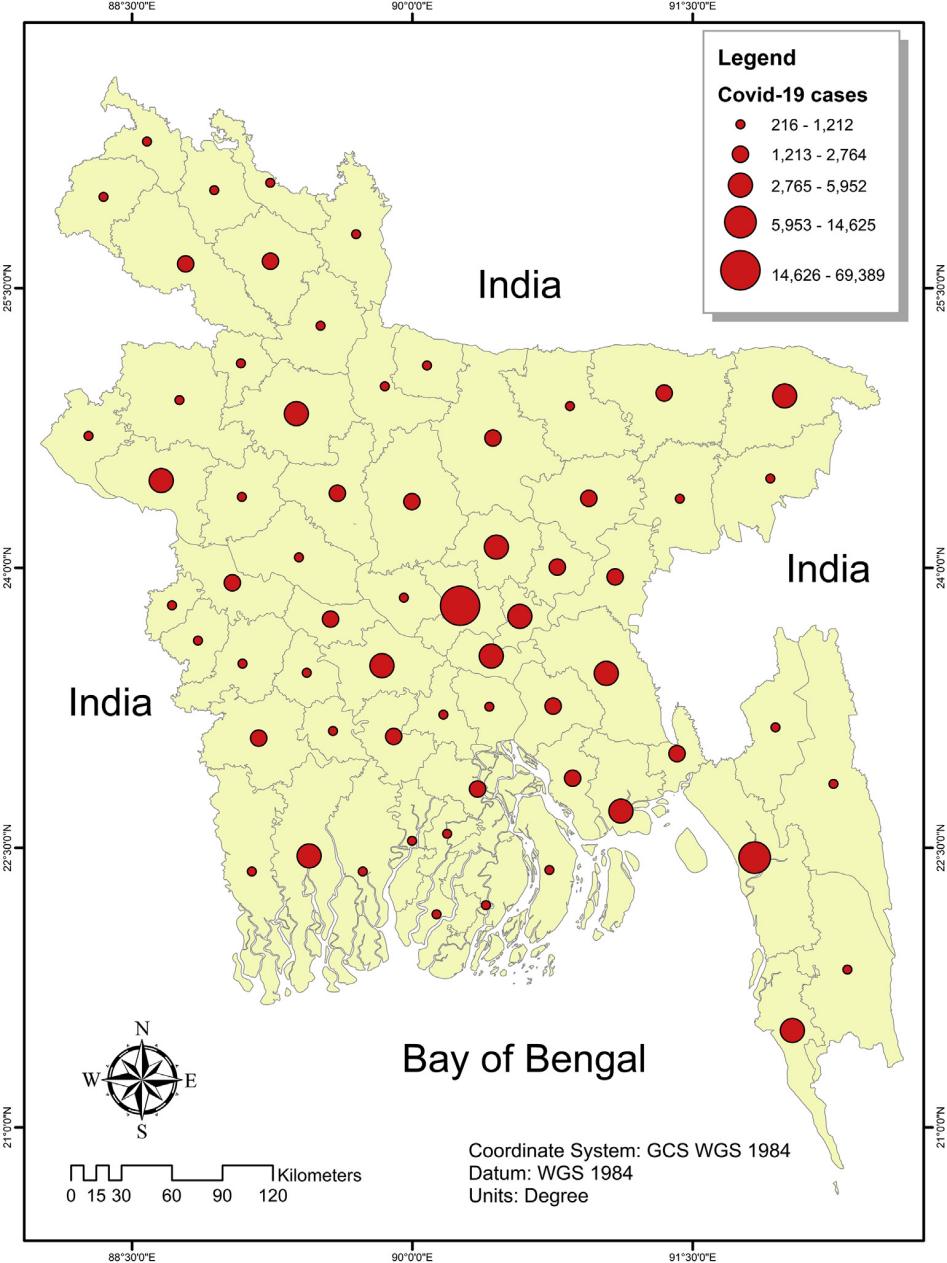
Distribution of COVID-19 cases at district level in Bangladesh as of 4 August 2020 (source: Author).

The Institute for Epidemiology, Disease Control and Research (IEDCR) is responsible for making the people in Bangladesh aware and informed about the spread and control of this novel coronavirus, test for COVID-19, and publication of the data of death toll, infected, and cured. Data on confirmed cases of COVID-19, collected from IEDCR (2020), till 4 August 2020 reveal spatially-concentrated cases (Figure 1) in the districts with international airports (e.g. Dhaka) and seaport (i.e. Chittagong).

Despite having better access to information, internet, and services in these major cities, they suffer higher rates of transmission (Figure 1). The cause is ignorance and denial of the possibility of transmission – a trait quite common among Bangladeshis, and Asians in general, whose cultures and religions tend to be fatalistic - that results from lack of information and awareness.

Pandemics cannot be stopped by Governments. Pandemics are too big and transcend borders, but their effects are created at the microbiotic level. Government are thus, at once, too big and too small to solve the problem alone. It is the action of billions of individuals in their daily lives that will ultimately limit, control and eliminate pandemic effects, especially before there is a reliable treatment protocol and vaccine. What we have seen, in Bangladesh and elsewhere, is that, despite, at times, draconian State action, people are not taking the actions necessary to limit or destroy this pandemic. In fact, many have no idea what their role is, what they should do and many more simply do not care.

Therefore, the hypothesis and argument of this paper is that, first, people must be aware – of the danger, of how they can prevent it. Then they must care. Then they can and will act. This paper focuses on the first step: awareness, because, as a race, we humans are not there yet. This research seeks to find out what causes people to be aware of the COVID-19 danger and how they can prevent it by their own action. Socio-economic factors are put forward as a hypothetical independent variable to test.

Until now, no study has been conducted to understand the roles of socio-economic factors in public awareness. Therefore, this research was about two basic questions about COVID-19 - (i) what is the role of socio-economic factors in creating public awareness? (ii) what are the socio-economic factors that can explain the level of public awareness at any point in time? In the process of investigating these questions, we discover the socio-economic factors influencing public awareness; thus, we propose a solution by emphasis on certain socio-economic factors to increase public awareness in order to control the transmission of the coronavirus disease.

## 2 Research methods

This research follows a mixed-method approach: it begins with a blend of an exploratory approach and observation methods from qualitative research to modelling from quantitative research. Questionnaire survey data helped to explain observations and made it possible to compare them with earlier observations; on the other hand, sometimes observations helped to understand and interpret the survey responses. The qualitative approach helps us to understand people's awareness about COVID-19 and to observe human attitudes. Quantitative methods explore the strength of factors that influence the respondents' awareness amid ongoing pandemic. Statistical regression techniques are used for derivation (i.e. explanation or prediction) of respondents' awareness level from observations of their socioeconomic statuses and behavioral factors. According to Hair et al. (2010) the acceptable sample size should be at least ten times of the variables. In this study, six variables were used, and the sample size was 274.

## 3 Data analysis and results

### 3.1 Data collection

This cross-sectional study is a part of non-funded academic research, which was conducted in May and June 2020. The target population is “adult Bangladeshis with access to internet”. This research utilized an online-based questionnaire to gather data on level of awareness and psychological state during the lockdown period of COVID-19.

**Figure 2:**
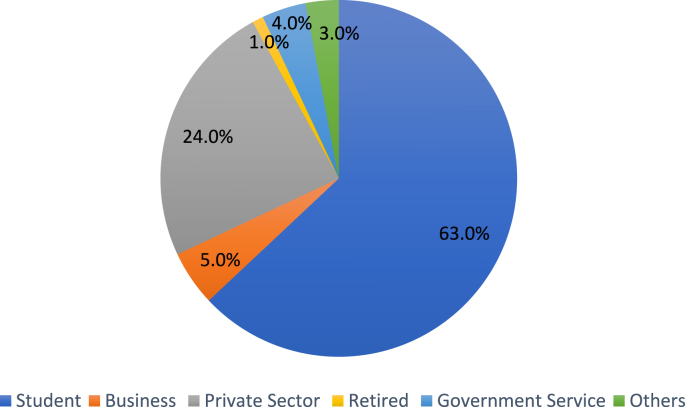
Occupational pattern of respondents.

Initially, a pilot survey was done. An online Google Form link was sent to 20 persons for the pilot survey through email and social media. The questionnaire (Google form) was comprised of 37 questions, including multiple choice questions, rank order questions using a Likert Scale and open-ended questions. Test-retest approach was taken for assessing the reliability of the questionnaire survey. Responses from the same individual were visually inspected instead of using statistical technique, e.g., correlation. As for the validity assessment for the questionnaire, face-validity method was followed and thus, the questionnaire was redesigned based on these assessments. The redesigned questionnaire considered the respondents' demographic aspects, physical state, before and during COVID-19, employment status, educational level, level of COVID-19 consciousness and psychological aspects. After necessary scrutinization and amendment per reviews from the pilot survey, the Google Form was made public and it reached 274 respondents. The 274 respondents, representing their households, portrayed information about 1,135 persons. single-stage cluster sampling techniques, combined with snowball sampling as some respondents forwarded the survey link to the acquaintances, was followed while distributing through e-mail and social networking platforms, e.g. Facebook. It is noteworthy that most of the respondents are young. A good number of responses originated from Dhaka because of better internet connectivity, cheaper cost of connection, availability of electronic gadgets, and Dhaka residents’ growing interest in participating in surveys due to the prevalence of Universities there. 63% of the respondents are students (Figure 2).

#### 3.1.1 Respondents’ consent

Respondents’ consent was taken before the survey, and all respondents have been informed that all information will be used for academic purposes only. Besides, the purpose and assurance of confidentiality of data collection were given to the respondents at the beginning of the survey.

### 3.2 Exploratory data analysis

#### 3.2.1 Demographic and household characteristics of the respondents

**Figure 3:**
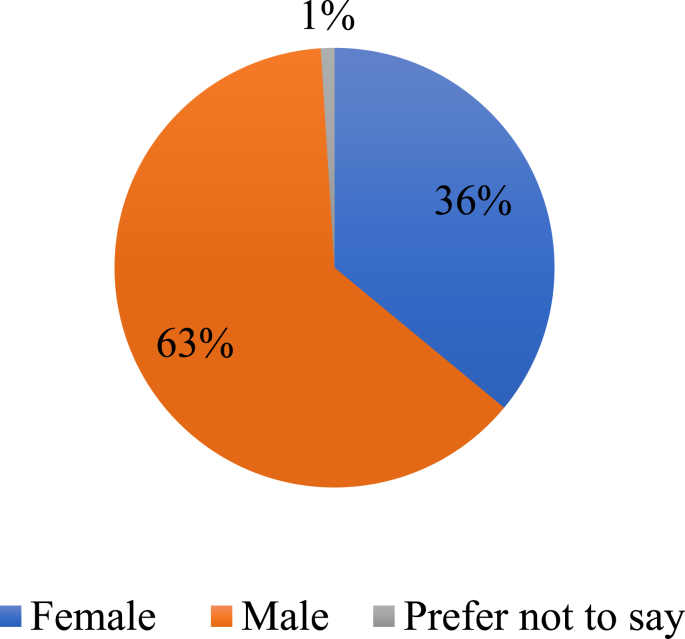
Gender distribution.

Most respondents were males (Figure 3) – an indication of gender disparity in access to internet and internet-based tools. Meanwhile, the mean age of 26 indicates the concentration of interest in cloud-based technologies among the young. Thus, information disseminated through the internet might reach smaller percentages of middle and older-aged persons.

The average household size of the respondent is 4 - and two-thirds of the families have one student in their household. It should be noted that internet access is limited in the rural regions and slum dwellers of the urban areas are neither interested in nor accustomed to online questionnaires The inclusion of such samples could increase the household size but the purpose of these questions are not to find out the household sizes: the purpose is to identify whether isolation is possible in a household. Therefore, omission of the possibility of inclusion of slum dwellers does not affect the study. Approximately 42% of families have at least one elderly (i.e. >65 years) person in the household. Half of the respondents own the residence they live in and the rest are tenants.

Number of bedrooms available in a household carries significance for minimizing the transmission of the virus within the home. A single-bedroom household with multiple dwellers increases the possibility of transmission and making the isolation (of the patient) nearly impossible. Even in two-bedroom households, isolation is still difficult. This study found that a large number of respondents are living in households of more than two bedrooms. Only 7 respondents (3%) are living in a single-bedroom apartments or studio apartments. It is very common for upper-class and even middle-class ho9useholds to keep a housemaid or at least take services from maids and hire personal drivers. Such services, along with security personnel and waste collectors, increase the possibility of transmission, as they can act as carriers. 30% of respondents’ families are found to be dependent on such services.

#### 3.2.2 Economic condition of the Respondent's family members

The average monthly income of the respondents’ families is BDT (Bangladeshi Taka) 57,253 ($673). Only 6% of respondents have household income less than BDT 15,000 ($175), while most of the households earn between 30,000 BDT and 60,000 BDT ($350 - $700). 1 out of 8 households has a monthly income of more than BDT 100,000 ($1176), which is 8 times higher than the per capita income of Bangladesh (World Bank n.d.).

The Government imposed worked from home to many State sector employees to provide the essential services. Currently, they have set a maximum of 25% of employees to be present in public offices, expecting the others to work from home in an attempt to minimize the spread of COVID-19 in the State sector workplaces.

**Figure 4:**
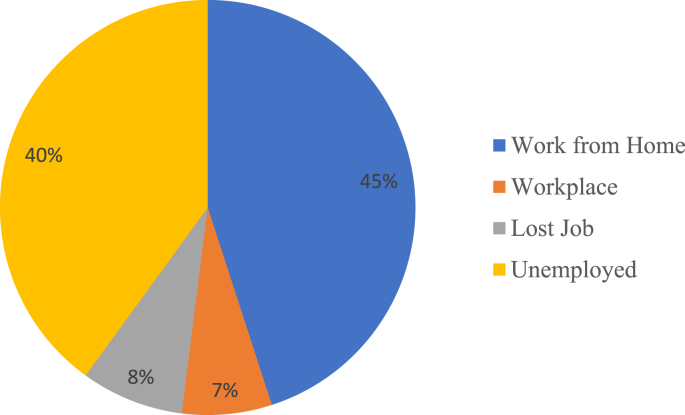
Employment Status of Respondents After Lockdown (in percentage).

A large percentage of respondents (45%) are working from home, while 7% are still going to their workplaces. Respondents lost their job due to lockdown or discontinuation of employer's business is reported by 8% of respondents (Figure 4). As most of the respondents are students, who were not employed prior to the lockdown the unemployment level of respondents is less than would be found in the general population. The layoff percentage doubles with exclusion of students from the samples. 75% of respondents earn from family properties as well as salary or business profits, reinforcing that this is an upper-class sample who do not depend on the sweat of their brows to eat: this is a result of the online survey, in effect drawing the sample from families who own computers and android phones, which are still luxuries in Bangladesh. The pandemic, however, adversely effected all sorts of income, earned and unearned.

During lockdown, the average amount of loss in income per family is reported as BDT 83,040 (US$977) and BDT 20,057 (US$236) within the first 2 months of lockdown. Compare this Bangladesh's per capita income of $1,909 in 2019 (‘Per capita income hits $1,909’ 2019). Economic loss and layoff have an impact on receptivity of the public to the COVID-19 awareness campaign. Although, “work from home” has had a positive impact in limiting the transmission of coronavirus and a large percentage of respondents (Figure 4) are doing this, joblessness and economic loss forces people out among other people to seek livelihood, helping to spread of the disease.

#### 3.2.3 Health of the respondents’ family members

Whether or not a person and his/her loved ones are healthy is a constant prime concern during a pandemic. Patients with pre-existing health conditions, *e.g*. diabetes, heart condition, are more likely to die from infection with COVID-19.

**Figure 5:**
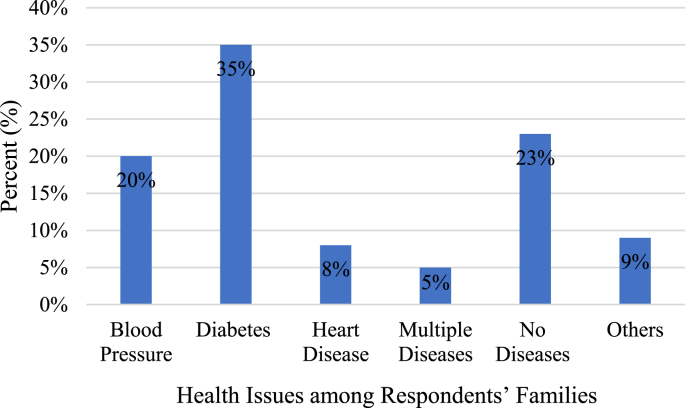
Health issues among respondents' families.

**Figure 6:**
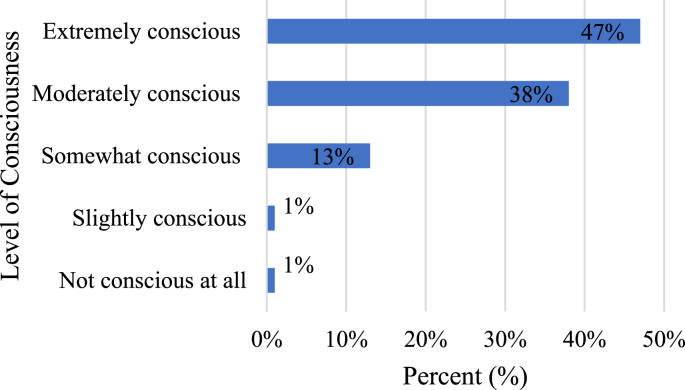
Level of consciousness among respondents.

Only one-fourth of respondents' families have no pre-existing health conditions (Figure 5). The most-vulnerable people are those who have multiple health issues: currently, 5% of respondents’ families have members with multiple health issues. Yet even these most-vulnerable families were not taking full precautions against COVID-19 and had wrong perceptions about how to do so (Figure 6). This alarming finding indicates failure of the awareness campaigns.

#### 3.2.4 Awareness and mental state during lockdown

Socio-economic factors alone cannot explain a lack of COVID-19 awareness. To understand the level of awareness, we must be aware of:

• The respondent's level of information about the danger;
• The respondents' confidence in and ability to process such information.

Information can be unintentionally false. Yet dissemination of information is imperative to reach the people whom we want to have it, even at the risk of disseminating false information.

We divided the process of investigating the state of information into two steps. First, we tried to understand availability of the test kits. Unavailability of test kits may result in a lower number of reported cases, which, in turn, creates a false impression of safety or that the pandemic is not serious. Then, we must find out where people are getting their information. Sources of information are important because knowing that is the key to reaching people. If we are disseminating information through channels that are not the ones most people use, they will never know about our message. Unfortunately, many of the most popular sources of information, like blogs and social media posts, are the least reliable. So, if we use them, our information might have to compete with a lot of rubbish to be heard and may not win that competition.

Formally, the lockdown was imposed from 27 March, after the first case of coronavirus infection had been reported in the first week of that month. A few weeks after the first flight arrival from Europe, carrying migrant workers and expatriates, the number of cases skyrocketed and the Bangladesh health system, within few days, could not handle the number of sick people who were rushing to them in panic... The number of unknown deaths and a concurrent epidemic of pneumonia cases created the image of a situation out of control. In many communities, no test kit for COVID-19 was available.

The Government was stung into action when a few high officials and a national Professor died of COVID-19 infection. The protests from the Doctors’ Association, demanding Personal Protective Equipment (PPE) and safety equipment, also put pressure on the Government through the media.

Social media has been playing a significant role and has been educating half of the population about preventative measures against COVID-19 infection (Hopman et al. 2020; Karasneh et al., 2020). The study found that:

• 2 out of 5 respondents were relying on television;
• 1 out of 4 were relying on online news portals;
• 23% of respondents were relying on social media

to get updates on COVID-19, including information on preventative and preparatory action, and reactive and mitigatory measures. The rest were relying on YouTube, printed newspapers, and other social media for updates. While social media can play a pivotal role in dissemination of information and in any awareness campaign, it is also a major source of rumors, fake news, and unverified information. As a result, the Bangladesh Government began to monitor online comments, promising to prosecute those who spread false prevent false rumors and fake news.

A question was formulated using Likert scale to find out the level of awareness of the respondents - 1 denotes less conscious and 5 denotes highly conscious. 47% of respondents were highly conscious and 38 percent were moderately conscious about COVID-19 (Figure 6). The percentages may seem higher than expected but, most of the respondents are students living in cities: thus, they were more-easily reached by the awareness campaigns. On the other hand, such a high level of awareness can be due to self-confidence. Self-confidence often creates negative impacts while processing information as a confident person might think no new information is needed. The self-confidence of respondents is supported by another finding: one-third of respondents are more concerned about their neighbors’ susceptibility to get infected than they are, whilst only 12% of the respondents think their family members are also likely to be infected.

**Figure 7:**
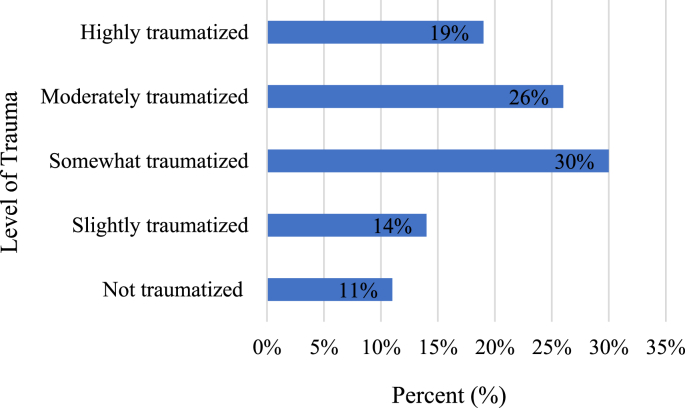
Trauma Experienced by Respondents (in percentage).

Taking care of mental health is equally as important as physical health, especially during a pandemic. Negative thoughts, depression and psychological trauma can not only affect mental health but also affects the intake, processing and storage of information. Therefore, a categorical variable was devised to inquire into the respondents’ mental state. Deterioration was found in 3 out of 5 respondents, with only 1 out of 5 remaining unchanged. 45 percent respondents felt a trauma on a Likert scale of 4 and 5 (Figure 7).

Approximately half of the urban respondents have an emergency plan if their family members get infected. Two-thirds of them have stocked a sufficient amount of staple foods and other necessary commodities for their family that cost around BDT 9,626 (US$113).

### 3.3 Binary and ordered logistic regression

Binary logistic and ordered logistic models were used to identify the socioeconomic variables which predict level of awareness of COVID-19 infection prevention and control. Logistic regression predicts the categorical dependent variable using multiple independent predictors (Peng et al., 2001). The nature of the dependent variable (level of awareness) is ordinal rather than continuous and proportional odds and the ordered logit model are widely used to predict the significant explanatory factors. The model applies the proportional odds assumption based on the Likert scaling. Here, the log of the dependent variable's odds is modeled as a linear combination of the independent variables.

(1) $$\text{Poorly,}\;\log\frac{{\text{P}}_{1}}{{\text{P}}_{2}+{\text{P}}_{3}+{\text{P}}_{4}+{\text{P}}_{5}}\text{,1}$$

(2) $$\text{Poorly}\;\text{or}\;\text{little,}\;\log\frac{{\text{P}}_{1}+\;{\text{P}}_{2}}{{\text{P}}_{3}+{\text{P}}_{4}+{\text{P}}_{5}}\text{,2}$$

(3) $$\text{Poorly,}\;\text{little,}\;\text{or}\;\text{moderately,}\;\log\frac{{\text{P}}_{1}+\;{\text{P}}_{2}+{\text{P}}_{3}}{{\text{P}}_{4}+{\text{P}}_{5}}\text{,3}$$

(4) $$\text{Poorly,}\;\text{little,}\;\text{moderately,}\;\text{or}\;\text{very}\;\log\frac{{\text{P}}_{1}+\;{\text{P}}_{2}+{\text{P}}_{3}+{\text{P}}_{4}}{{\text{P}}_{5}}\text{,4}$$

Here, the levels of awareness are expressed as "poorly", "little", "moderately", "very", and "extremely" by, respectively, p1, p2, p3, p4, p5.

Akaike Information Criteria (AIC) is analogous to adjusted R^2^; it is the measure of fit which improvised the model for the number of independent variables for finding minimum AIC value. AIC of the ordinal logit model compares the model derived by step AIC function to get the best match. To predict such multi-class ordinal variables, proportional odds logistic regression is considered as a useful technique (Williams 2016).

For another model, the outcome variable is transformed into binary form to estimate the explanatory factors on binary outcomes (Washington et al., 2020; Ma et al., 2020). The level of awareness is converted to a dummy variable based on the values of the Likert scale. The dummy dependent variable uses a single regression equation that omits the equations from the dependent variable's sub-groups. The binary logit model predicts the odds of a successful outcome compared to unsuccessful ones.

(5) $$\text{Logit}\;\text{P}=\text{Pr}\left({\text{Y}}_{i}=1|{\text{X}}_{i}={\text{ x}}_{i}\right)=\frac{\text{exp}\left({\text{β}}_{0}+{\text{β}}_{1}{\text{x}}_{i}\right)}{1+\text{exp}\left({\text{β}}_{0}+{\text{β}}_{1}{\text{x}}_{i}\right)}$$

or,

(6) $$\text{Logit }\left(\text{Y}\right)=\text{ log }\left(\frac{{\text{Y}}_{i}}{1-{\text{Y}}_{i}}\right)$$

(7) $$\;={\text{β}}_{0}+{\text{β}}_{1}{\text{x}}_{i}$$

(8) $$\;=\;{\text{β}}_{0}+{\text{β}}_{1}{\text{x}}_{i1}+\ldots \;+{\text{β}}_{1}{\text{x}}_{ik}$$

${\text{Y}}_{i}$ = 1 if the level of awareness is 5 in Likert scale in the datum i
${\text{Y}}_{i}$ = 0 if the level of awareness is in between 1-4 Likert scale in the datum
${\text{X}}_{i}$ = (${\text{X}}_{1}$, ${\text{X}}_{2}$, ..., ${\text{X}}_{k}$) are a set of independent/explanatory variables. ${\text{x}}_{i}$ is the observed value of the explanatory variables for datum i.

#### 3.3.1 Dataset preparation

The responses were stored automatically in Google sheets but, for convenience, the dataset was converted to CSV format and prepared in Microsoft Excel. All incomplete and duplicate responses were deleted from the raw dataset. Exploratory analyses were performed to determine the respondents' demographic characteristics and select the potential predictors for modeling. Numeric and continuous data were presented as means with percentages and categorical data were expressed as frequencies with percentages.

#### 3.3.2 Outcome variables

Level of awareness about COVID-19 was chosen as the dependent variable for regression analysis. The level of awareness was obtained from the respondents on a scale ranging from 1 to 5, where 1 denotes a complete lack of awareness and 5 denotes extremely aware about COVID-19 dangers and prevention protocols. The value of 2 on a Likert scale does not mean that a person is two times more aware than a person for whom 1 has been answered from the questionnaire: ordered data cannot be measured by a ratio scale or simple mathematical equation. Ordinal modelling techniques are necessary to predict outcomes and explanatory factors in such a case.

A dummy dependent variable was generated using a Likert scale where level 5 denotes 1 and all other levels denote 0. Thus, a binary logit model was developed using the formulated dummy variable and compared with the output using an ordinal logit model.

#### 3.3.3 Independent variables

During a lockdown, people only go outdoors for livelihood or other necessary reasons: few go outdoors being ignorant of the lockdown and of the consequences. Frequency of going outdoors and interval times can, thus, be an explanation of the awareness level of respondents. It assumes, highly-aware conscious respondents remain indoors more than 2 weeks and unaware ones go outdoor once in each week. As the 14-day incubation period of coronavirus is an important factor for COVID-19 prevention people, who went outdoors 14 days or more, were put into a one category and the rest were put into another category. A dummy variable was encoded: where there was at least 1 departure from the house the last 14 days this was coded as 0 and a departure in the last 15 days was coded as 1.

Another way to explain the level of awareness is the number of stored products for the pandemic, as it indicates sensing the “expectation of shortage” - a possible effect of pandemic. Stockpiling is a common phenomenon for any emergency and this pandemic is no exception. People bought and stored necessary items during the pre-lockdown phase to be able to remain indoors during lockdown and to limit the frequency of outdoor activities. Frequency of outdoor activities is considered as a potential predictor as it assumes, stockpiling has a positive correlation with awareness regarding COVID-19. Therefore, the number of stored products were converted to dummy variables - number of stored products more than 4 was coded as 1 and less than 4 was 0. Use of masks in outdoor activities was another binary independent variable to explain the level of awareness of COVID-19.

People get traumatized during pandemics for many reasons - some may encounter distressing and negative news from social, broadcast, or print media; someone may receive upsetting news of close persons getting infected. Such stimuli create an adverse momentum in their psychology, leading to an asymptomatic, subconscious trauma. Level of trauma was recorded on a Likert scale where 0 indicates less traumatized and 5 show highly traumatized mental state. In addition, income was also taken as independent variable, as the those in the higher income group believe they have better access and resources to survive during pandemic.

## 4 Findings and discussions

Social awareness is very important during a pandemic. Improved habits of personal hygiene, restricted movement and level of awareness can significantly reduce the possibility of infection. The level of awareness is dependent on several factors and this study try to identify the underlying socioeconomic factors related to awareness. Here the level of awareness is measured in order through Likert scale, which is used to construct the ordinal and binary logistic models. 5-point scale is easily perceivable by the general participants and it is widely used both in qualitative and quantitative research.

Table 1 shows the results of two different models and the difference between these two models is the technique of their estimation process. The possible values for the outcome variables for binary logistic (model 1) is between 0 and 1, on the other hand in the ordinal model, the values of outcome variable ranges from 1 to 5.

**Table 1:** Binary logistic and ordinal logistic regression models for assessing level of awareness towards COVID-19.

Dependent variable		
	Level of awareness Binary logistic (Model 1)	Level of awareness Ordinal logistic (Model 2)
Number of stored products	0.789∗∗∗ (0.287)	0.915∗∗∗ (0.274)
Home Departures	0.567∗∗ (0.264)	0.845∗∗∗ (0.247)
Mask (never used)	-14.087 (613.754)	-44.752∗∗∗ (0.000)
Mask (irregularly)	-1.242∗∗ (0.592)	-1.015∗∗ (0.432)
Mental trauma	0.234∗∗ (0.109)	0.267∗∗∗ (0.102)
Income (15K–30K)	1.111∗ (0.633)	0.786 (0.534)
Income (31K–60K)	0.813 (0.600)	0.184 (0.496)
Income (61K–100K)	0.341 (0.621)	0.041 (0.512)
Income (100K+)	1.301∗∗ (0.662)	0.727 (0.567)
Constant	-2.145∗∗∗ (0.681)	
Observations	274	274
Log Likelihood	-172.225	
Akaike Inf. Crit.	364.450	

*Note:* ∗p < 0.1; ∗∗p < 0.05; ∗∗∗p < 0.01.

A binary logit model was simulated for better AIC score and the minimum value was selected as a best match. The confusion matrix shows that the accuracy of the model is 43.7%.

**Figure 8:**
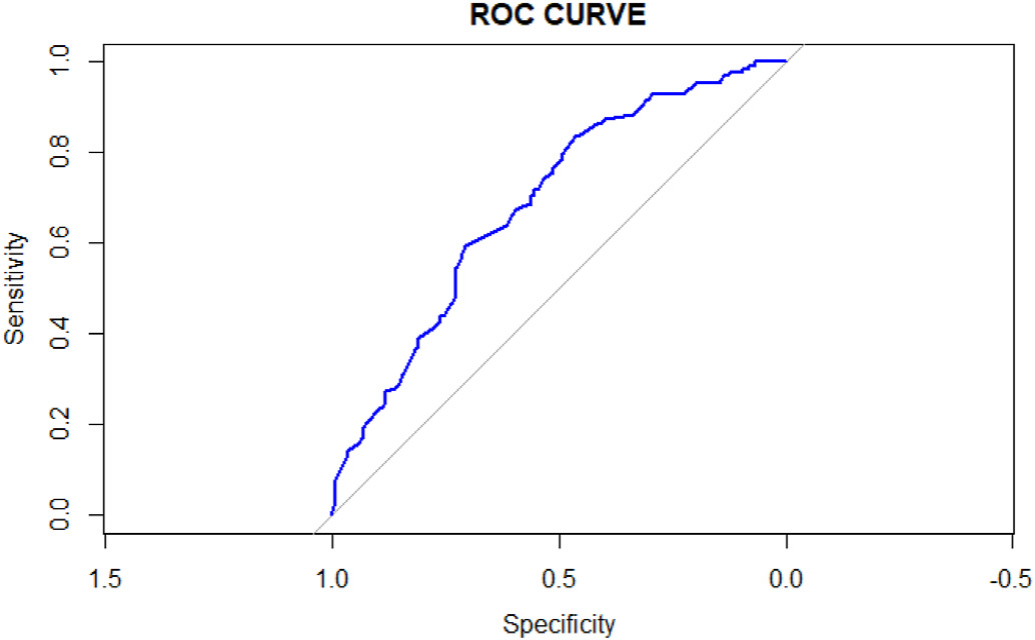
ROC curve.

Receiver Operating Characteristics (ROC) explains the performance of the model by evaluating sensitivity and specificity. Considerable area under the ROC curve portrays better accuracy and better predictability of the model. The area under the curve of the devised model is 0.688 (Figure 8) (where a value of 1 indicates a perfect model).

Table 1 compares the results of two different models. The dependent variable of the first model is binary and in the latter model it is an ordered variable. In the binary logit model, the number of stored products, departures from the home, use of mask, trauma and income show statistically significant results. This suggests that the respondents who stored more items, stayed at home more often, wore a mask outside, suffered trauma and had higher incomes are more likely to be aware about the COVID-19 pandemic. This confirms the hypotheses as explained in the previous section of this paper. These variables receive similar significant results in the ordinal logistic model as well.

Both models indicate that respondents who were outdoors at least once in the last 14 days are less conscious than those who remained indoors for the whole time. The result shows, respondents used mask irregularly are significantly less conscious than those who used it regularly; those who were never used mask during pandemic are less conscious than occasional users. This estimation is not statistically significant in the binary logit model but highly significant in the ordinal logit model.

Very often, mental trauma compels people to be overly cautious. Both models show that the respondents who are more traumatized are more conscious about COVID-19. For the income of the respondents, result indicates higher income groups and lower-middle income groups have higher levels of awareness than other groups. But income does not show any significant result in the ordered logit model.

Table 2 shows that large numbers of stockpiles, less departures from home, more use of masks, and mental trauma are significant predictors. The odds ratio of significant variables indicates more plausible influence on awareness levels than other variables, i.e. they are better predictors. In the ordinal logit model, the threshold coefficients 1|2, 2|3, and 4|5 are significant. Particularly, the likelihood of receiving a 1, 2, 3, or 4, as opposed to a 5, is highly significant and has a higher odds ratio.

**Table 2:** Odds ratio of binary logistic and ordinal logistic model.

Predictors	Level of awareness Binary logistic (Model 01)		Level of awareness Ordinal logistic (Model 02)	
	Odds Ratios	std. Error	Odds Ratios	std. Error
(Intercept)	0.12 ∗∗	0.68		
Num of stored products	2.20 ∗∗	0.29	2.50 ∗∗∗	0.27
Home Departures	1.76 ∗	0.26	2.33 ∗∗∗	0.25
Mask (never used)	0.00	613.75	0.00 ∗∗∗	0.00
Mask (used sometimes)	0.29 ∗	0.59	0.36 ∗	0.43
Mental trauma	1.26 ∗	0.11	1.31 ∗∗	0.10
Income (15K–30K)	3.04	0.63	2.19	0.53
Income (31K–60K)	2.25	0.60	1.20	0.50
Income (61K–100K)	1.41	0.62	1.04	0.51
Income (100K+)	3.67 ∗	0.66	2.07	0.57
1|2			0.02 ∗∗∗	1.14
2|3			0.05 ∗∗∗	0.80
3|4			0.94	0.57
4|5			7.65 ∗∗∗	0.58
Observations	274		274	
R2 Tjur	0.115		0.235	

*Note:* ∗p < 0.05 ∗∗p < 0.01 ∗∗∗p < 0.001.

Tjur's R^2^ values in Table 2 shows that, for this dataset, predicting level of awareness through the binary model is less efficient than through the ordered logit model, although the values of Tjur's R^2^ for both models were low because of limited numbers of observations and presence of heterogeneity in the dataset. Increasing homogenous observations could result in a better prediction model. Tjur's R^2^ values, as they are here, suggest that the predictive power of the two models must be described as limited.

The predictors of level of awareness towards COVID-19 will help us to understand the influencing factors that affect human behavior during lockdown. The demographic characteristics of the respondents are not unique but behavioral and cultural patterns are quite similar. Although the level of awareness also depends on many unexplained and unobservable exogenous factors, this study has revealed a few of them in an attempt to explain the level of awareness during COVID-19.

It is notable that most of the respondents think that they have a higher level of awareness than their behavior indicates. Many of them also think their neighbors are more susceptible to transmission than themselves and their family members are – a lack of trust in their neighbors as well as overconfidence in themselves.

All of these perception factors subtly indicate a lack of awareness about the facts relating to COVID-19 transmission. Overconfidence skews perception, makes the subject selective about receiving/perceiving/believing information and is both the cause and effect of lack of awareness in a vicious cycle: like the world leaders, in countries like America and Brazil, who announced to their people that coronavirus infection is just “a little flu” and caused millions (including themselves!) to be infected when they tuned out COVID-19 information. Overconfidence often leads to poor choices or decision and to underestimating the actions taken by others (Hackston 2019). The deadly part of overconfidence during the pandemic has been when it caused people to grossly underestimate the probabilities of COVID-19 infection and death and therefore to decide to act in dangerous ways.

Secondly, coronavirus infection prevention depends on many exogenous factors: education, experience, age and so on. For example, young people are highly-likely to pass the disease to older people, who are more-likely to die from it, but the young people cannot see the need for prevention measures once they understand that they are highly-unlikely to become symptomatic and even less likely to die from infection. Awareness must support a pre-existing sense of moral obligation to one's self, one's close persons and to strangers whom one encounters. It is that sense of moral obligation that induces people to take action to protect themselves and others from the danger of which they become aware. Moral obligations have been, throughout history, notoriously difficult to impose and enforce using legislative and administrative power: obligations cannot be injected and what the State demands outside has no effect if there is no sense of obligation inside. Knowledge without a sense of moral obligation induces no action but hangs in the air like coronavirus itself.

Compounding the problem is that the lockdowns virtually destroyed the economy everywhere they occurred. Morally-degraded people in a condition of economic crisis are not the best people to rely on to help in preventing the spread of coronavirus. Ending the lockdowns presented the opportunity to convince people to use more sustainable measures of prevention – social distancing, masks, handwashing, testing/tracing, etc. – as they began to recover economically and became less “dog-eat-dog” in their thinking. Governments are only starting to understand this: too often, they have rocketed between the extremes of lockdown and nothing, month-by-month. They need to choose “the middle way” with a long-term focus.

Hygiene is a powerful weapon to fight COVID-19 infection. Adequate knowledge about hygiene is very important from public health perspective. Hygiene practice of every individual is a clear indication of high-level awareness. As Bangladesh has never experienced such a pandemic before, it is difficult for the masses to perceive the consequences of COVID-19 before it infects them or their family members. The psychology of behavior is rooted in learning from experience. However, in a developing country like Bangladesh, with an overcrowded population of 160 million, the existing infrastructure is hardly good enough to be effective without supplementation by individual action. Therefore, the stakeholders must understand the influence socio-economic factors and act upon them to change mass awareness and, thus mass behavior. That is the ultimate desired output of this research.

## 5 Conclusion

Awareness is considered as the most effective non-structural mitigation measure to fight against any unprecedented event. Promoting awareness is not a difficult task if it can be done is a systematic manner. Socio-economic and psychological factors often play a pivotal role to predict the level of awareness. Thus, action to create awareness and turn it into action must begin with action on these socio-economic and psychological factors.

This paper has used an online questionnaire survey to collect data, supplemented by snowball sampling as some respondents forwarded the survey link to the acquaintances. Modelling was used to identify the significant socio-economic factors which influence public awareness. From past pandemics, it is perceived that, the success of strategic plans to decrease the rapid transmission of a highly-infectious disease partially depends on people having accurate perceptions of personal and societal risk factors (Dryhurst et al., 2020; de Bruin and Bennett 2020). Such perception is built up when accurate and brief messages are conveyed. These must be prompt, precise and culturally suitable messages and they will increase the public's confidence in Government's ability to control the pandemic (Hua and Shaw 2020). Such messages also make people keen to take recommended precautionary measures (Hopman et al. 2020). However, significant factors are needed to be identified beforehand so that Government is able to devise the message that is socially, culturally and economically suitable for the target audiences. Therefore, ensuring public awareness at the time of a serious public health crisis can significantly improve the situation within a shorter period (Alam et al., 2020).

The level of awareness about COVID-19 is significantly dependent on socio-economic, behavioral, and psychological factors: notably, income, outdoor activities, use of masks, number of stored products, and mental trauma. The findings of this research suggest that we must ensure steady income and places for physical activities and recreation, in order to prevent the transmission of the disease by making citizens aware of the safety precautions needed.

However, the narrow socio-economic basis of the sample – wealthy students in the capital who have computers – is a shortcoming of this study. Another shortcoming of this paper is that it focused on primary, original data collection, so data from State reports and documents are not always included: had they been included, some results may have changed.

Future research can explore strong predictors having higher R-square values that can better explain the total variance of the dependent variable. Better modelling techniques and machine-learning algorithms could generate unbiased and consistent predictors.

## Declarations

### Author contribution statement

Md Rifat Hossain: Conceived and designed the experiments; Performed the experiments; Analyzed and interpreted the data; Contributed reagents, materials, analysis tools or data; Wrote the paper.

Salit Chakma: Analyzed and interpreted the data; Contributed reagents, materials, analysis tools or data; Wrote the paper.

Farah Tasnim, Zuairia Zahra: Conceived and designed the experiments; Performed the experiments; Wrote the paper.

### Funding statement

This research did not receive any specific grant from funding agencies in the public, commercial, or not-for-profit sectors.

### Data availability statement

Data will be made available on request.

### Declaration of interests statement

The authors declare no conflict of interest.

### Additional information

No additional information is available for this paper.
